# Effects of Filler Anisometry on the Mechanical Response of a Magnetoactive Elastomer Cell: A Single-Inclusion Modeling Approach

**DOI:** 10.3390/polym16010118

**Published:** 2023-12-29

**Authors:** Timur A. Nadzharyan, Elena Yu. Kramarenko

**Affiliations:** Faculty of Physics, Lomonosov Moscow State University, 119991 Moscow, Russia; nadz@polly.phys.msu.ru

**Keywords:** magnetoactive elastomers, magnetorheological elastomers, theoretical modeling, ferromagnetic filler, stiffening effect, magnetorheological effect, anisometric particles

## Abstract

A finite-element model of the mechanical response of a magnetoactive elastomer (MAE) volume element is presented. Unit cells containing a single ferromagnetic inclusion with geometric and magnetic anisotropy are considered. The equilibrium state of the cell is calculated using the finite-element method and cell energy minimization. The response of the cell to three different excitation modes is studied: inclusion rotation, inclusion translation, and uniaxial cell stress. The influence of the magnetic properties of the filler particles on the equilibrium state of the MAE cell is considered. The dependence of the mechanical response of the cell on the filler concentration and inclusion anisometry is calculated and analyzed. Optimal filler shapes for maximizing the magnetic response of the MAE are discussed.

## 1. Introduction

Magnetoactive elastomers (MAEs) are composite materials that consist of a flexible polymer matrix and small magnetic particles, either micro- or nanometer in size, that are embedded in the matrix [1,2,3,4,5,6,7,8,9,10,11]. These materials fall into the category of so-called smart materials due to their ability to undergo significant changes in their physical properties or overall behavior when exposed to controlled magnetic fields. Of particular interest is the effect the magnetic field has on their deformational and viscoelastic properties, as well as various electromagnetic properties, such as magnetization reversal curves, magnetic permeability, electrical conductivity, and dielectric permittivity [8,9].

One notable phenomenon exhibited by MAEs is the magnetorheological (MR) effect. This phenomenon causes significant changes in the storage and loss moduli of these materials when exposed to external magnetic fields, resulting in changes in their shear behavior. This distinctive behavior has led to MAEs also being referred to as magnetorheological elastomers [4]. The MR effect is the focus of much work on MAE properties and serves as the basis for several important practical applications of MAEs, namely, soft robotics, vibration control, haptic feedback devices, pressure sensors, wearable devices with tunable pressure for biomedical purposes, etc. [12,13,14,15,16,17,18,19,20,21]. The ability of MAEs to rapidly change material properties on demand makes these materials valuable in various fields where controllable mechanical responses are needed.

The field of MAE studies has been experiencing active development for the past two decades. Several comprehensive reviews are available that focus on fabrication, characterization, and applications of these materials [2,4,5,6,7,8,9,22]. The state of theoretical studies of MAEs is discussed at length in [7,11,23,24]. The interest surrounding MAEs is driven by their potential applications in civil engineering as vibration dampers, remotely controlled switches, and noise control systems [25,26,27,28,29,30,31], in soft robotics as actuators [13,14,32,33,34], in entertainment as parts of haptic feedback devices [15,35,36,37], in biomedical devices as adaptive compressors and seals [38,39,40,41,42], in communication systems as microwave and radio frequency filters [43,44,45], and in automobile production as active braking systems [46,47,48]. Historically, the majority of both fundamental and applied research concerning MAEs has centered on harnessing the bulk properties of these materials. However, recent studies have unveiled MAEs as exceptionally promising materials for swiftly and reversibly manipulating various surface properties. This particularly involves characteristics like wettability [49,50,51,52], surface roughness [50,53], adhesion [54,55], and friction [56]. This newfound insight opens the door to novel applications of MAE-based intelligent surfaces across diverse domains. Examples include droplet-based microfluidics [57,58,59,60,61], devices for transporting and distributing liquids [52,62,63,64], fog harvesting technologies [64,65], and locomotion mechanisms for soft robots [66,67].

The primary underlying factor responsible for magnetic sensitivity of MAEs is believed to stem from the restructuring of the ferromagnetic filler particles or, in other words, their mutual rearrangement under the influence of external magnetic fields, involving shifts in their relative positions or, equivalently, alterations in the microstructure of the composite material [8]. This phenomenon draws parallels to the behavior observed in magnetorheological fluids, where particles align themselves along magnetic field lines, forming elongated clusters [68]. A significant reorganization can occur only when the polymeric matrix is soft and possesses a shear modulus that falls below 100 kPa [9].

Despite significant progress in describing the behavior and properties of MAEs that has been made over the last decade, a comprehensive theoretical framework capable of explaining and predicting the diverse array of attributes and behaviors exhibited by mechanically pliable MAEs has yet to be formulated. This challenge is attributed to the magnetomechanical coupling, substantial variability in material composition, and the necessity of accounting for the nonlinear properties of the constituent materials. The magnetization of ferromagnetic particles displays a nonlinear relationship with the internal magnetic field, and, in the case of hard magnetic particles, the influence of magnetic hysteresis cannot be disregarded. When ferromagnetic particles are moved (translated and/or rotated) within an applied magnetic field, the encompassing polymeric matrix undergoes deformation. Magnetic interactions—both between individual magnetized particles and between each particle and the external magnetic field—as well as elastic forces originating from matrix distortions compete when an MAE specimen is subjected to a magnetic field. In a broader context, elastomer matrices exhibit nonlinear viscoelastic properties, further complicating the theoretical description. Clearly, the wide array of synthesis conditions, material compositions, specimen shapes, and excitation parameters (including magnitude, direction, and temporal behavior of the external magnetic field) necessitates a comprehensive, multi-scale model for MAE materials.

Existing continuum models cannot describe filler restructuring in MAEs, which plays an important role in defining their properties [69,70,71,72]. The continuum approach is used for macroscopic description of the composite behavior. The works [73,74] showcased an invariant-based analytical framework previously developed by the same authors for dielectric elastomers applied to MAEs with an effective isotropic response. Macroscopic deformation of MAE samples containing iron and ferrofluid particles was studied using the homogenized free energy of the material. Micro- and mesoscopic modeling of MAEs that explicitly considers the presence of microscopic filler particles and their magnetic interactions has advanced significantly in the recent years [75,76,77,78,79]. However, the vast majority of microscopic theoretical studies focus on MAEs with spherical filler particles. The influence of the particle shape on the material properties has not been sufficiently explored. Recently, several experimental works that focused on the response of polymer composites containing elongated and platelet filler particles have been published [80,81,82,83]. It was shown that the magnetorheological effect can be enhanced using anisometric filler. Furthermore, the work [84] proposes to use anisometric filler particle as a probe for microrheology. It follows that a model with filler particle shape variability taken into account has value for describing and analyzing new complex materials.

In this research, we consider the influence of filler inclusion shape on its displacement in a magnetic field and the resulting change in the properties of magnetoactive elastomers on the scale of a dense cluster of magnetic particles or a single ferromagnetic inclusion. The relative simplicity of this approach allows us to focus on different elementary excitations of the unit cell (representing an average volume element per inclusion) and to estimate how each of them affects the structural and mechanical response of the composite material to external load for the case of low filler concentration values. The cell boundary conditions determine the general internal structure of the composite material and the influence surrounding cells have on a selected cell in the proposed model. A force of mechanical nature can be applied to the cell boundary, and a force of either mechanical or magnetic nature can be applied to the ferromagnetic inclusion inside the cell. The inclusion is affected by an external magnetic field and moves inside the polymer medium if the magnetic part of the total energy dominates over the mechanical energy stored in the cell. The inclusion’s displacement can be a result of the rotation of its magnetic moment in order to align it with the external field’s direction, the center of mass of the inclusion moving in non-uniform magnetic field, and the cell itself deforming under the influence of external mechanical forces. The last factor can be attributed to the movement of other inclusions in the material deforming the matrix and creating forces acting on the boundary of the cell under consideration. It follows that the three main mechanical processes in the cell are as follows: inclusion rotation, inclusion translation, and the deformation of the cell. The method used in this work combines FEM simulations for the mechanical part of the problem and minimization of an energy functional corresponding to a magneto-mechanical cell problem. To the best of our knowledge, this approach has not been used to consider MAEs with anisotropic fillers, as most of the currently available research focuses on composites containing spherical particles.

The paper is organized as follows: in Section 2, the modeling process and the general modeling assumptions are explained for an MAE cell containing a single inclusion. In Section 3, verification of the proposed model is carried out using two different theoretical approaches. The results of FEM simulations of the mechanical response of the cell to different mechanical excitations are presented and analyzed in Section 4. The influence of uniform external magnetic fields on the state of the cell is discussed in Section 5. Finally, the results are summarized in the concluding section.

## 2. The Modeling Process

**Figure 1:**
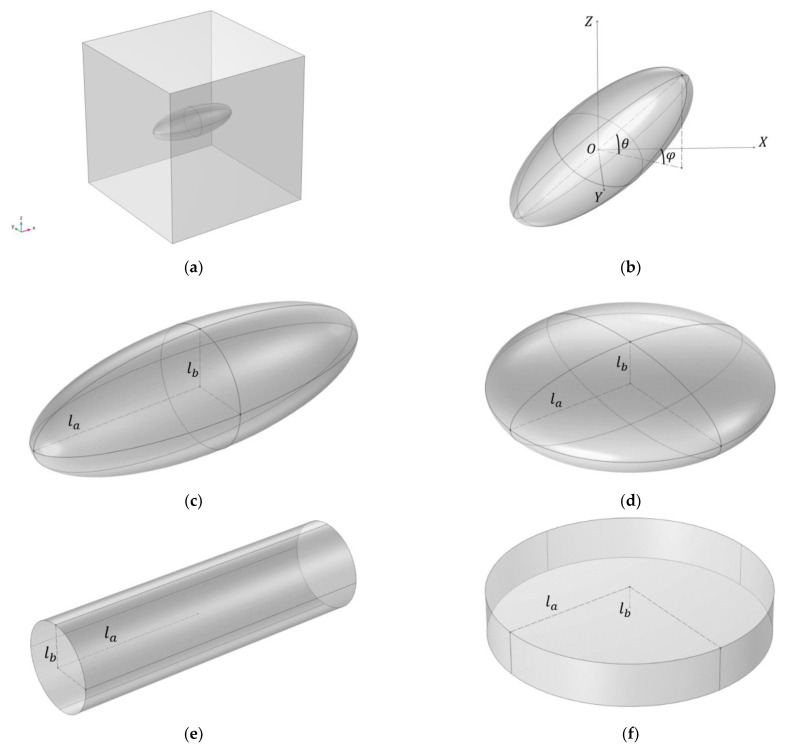
The geometrical setup of the model. (**a**) A cubic cell containing a single ferromagnetic inclusion; (**b**) spatial orientation of the inclusion; (**c**) prolate spheroid inclusion; (**d**) oblate spheroid inclusion; (**e**) rod-like inclusion; (**f**) disk-like inclusion.

Here we study the response of a composite volume element containing a single inclusion embedded in polymer network (Figure 1). The size of the inclusion is assumed to be large compared to the average distance between crosslink points in the polymer matrix.

### 2.1. General Outline

Let us consider the inclusions with both shape and magnetic anisotropy. The two main basic shapes are cylindrical and ellipsoidal. We consider an inclusion that possesses an axis of symmetry in this study. A cylindrical inclusion can have different ratios of its height to the diameter of its base (for example, greater or less than unity—a rod or a disk). An ellipsoidal inclusion is considered to be either an oblate or a prolate spheroid. One of the most important parameters characterizing the shape of the inclusion is the anisotropy parameter r that is defined as a ratio of two of the inclusion’s linear dimensions: $r=l_{a}/l_{b}$ (see Figure 1c–f). For example, for an ellipsoidal inclusion, the parameter r is equal to the ratio of the longer axis length to the shorter axis length. Assuming that the direction of the external magnetic field is the same as the OZ axis, the inclusion’s position inside the cell is fully described by its center of mass radius vector $\vec{\delta }$ and two angles: the angle between the inclusion’s symmetry axis and the XOY plane (θ) and the angle between the OX axis and the projection of the inclusion’s symmetry axis on the XOY plane (φ) (see Figure 1b).

Modeling of the unit cell is based around solving the boundary value problem in the weak form for the inclusion movement in a cell using the finite-element method (FEM) and energy minimization. The inclusion is placed at the origin point of the cell, and by default $\theta_{0}=0{}^{\circ}$ and $\varphi_{0}=0{}^{\circ}$ (as it is shown in Figure 1a). All linear dimensions are set to be dimensionless, and the unit cell is modeled as a cube with the edge length that depends on filler volume concentration c. Thus, both the size and the volume of the modeled cell are defined by the parameter c. The diameter of the spherical inclusion (the case of r=1) is taken as a unit of length in the dimensionless formulation. The volume of the inclusion is kept constant in all simulations. It follows that the linear dimensions of the anisotropic inclusion are defined by the anisotropy parameter r.

The total energy of the cell in the presence of external magnetic field $\vec{H}$ can be expressed as the sum of the magnetic energy of the cell $W_{magn}$ and elastic energy contribution $W_{el}$:

(1) $$W_{cell}=W_{magn}+W_{el},$$

After the dependence of $W_{el}$ on inclusion rotation and displacement of its center of mass as well as cell deformation is calculated using FEM simulations, the cell state can be obtained by minimizing the energy functional $W_{cell}$ with respect to parameters that characterize the position of the inclusion and its magnetic moment. The cell state is completely determined in this approach as mechanical FEM simulations provide us with the stress and strain distribution dependence on the inclusion displacement parameters θ, φ, and $\vec{\delta }$. It is important to note that $W_{el}$ can be calculated independently of the nature of the force acting on the inclusion as it represents the mechanical reaction of the cell to an external stimulus. This method of MAE description was established and used in [70] and allows for separation of the mechanical component of the problem from the magnetic component. $W_{el}$ determines the mechanical response of the cell to inclusion displacement or cell deformation and $W_{magn}$ determines the forces that lead to this displacement/deformation. While this model does not allow us to calculate macroscopic material properties without a corresponding homogenization procedure, studying the response of an MAE cell allows us to obtain the trends of filler restructuring and to evaluate the effect of the filler particle shape on this process. We first focus on the mechanical part of the problem and study how inclusion size and shape as well as filler concentration influence the cell response to different types of mechanical excitations. Those types are the following: inclusion rotation, inclusion translation along different directions, and uniaxial cell deformation.

### 2.2. Elastic Energy

The polymer surrounding the inclusion is represented by a hyperelastic medium with the neo-Hookean elastic strain energy density function:

(2) $$\psi =\frac{G}{2}\left(I_{1}-3\right)-Gln\left(J\right)+\frac{\lambda }{2}{ln}^{2}\left(J\right),$$

Here, G is the shear modulus of the medium, λ is the Lame parameter, $I_{1}$ is the first invariant of the right Cauchy–Green tensor, $J=det\hat{F}$ and $\hat{F}$ is the deformation gradient tensor that represents the relationship between the reference and current configurations of the deformed system. The elastic energy of the cell can then be expressed as follows:

(3) $$W_{el}=\int_{V\backslash V_{i}}\psi dV=W_{el}\left(\theta ,\varphi ,\vec{\delta }\right),$$

Here, V is the cell volume and $V_{i}$ is the inclusion volume. The mechanical excitations in the polymer matrix are caused by the movement of the inclusion, so the elastic energy is equal to zero when the inclusion movement parameters are equal to their respective initial values: $\theta =\theta_{0}$, $\varphi =\varphi_{0}$, and $\vec{\delta }=0$. The mechanical energy of the cell stored as a result of inclusion movement also depends on the geometric and mechanical parameters of both the cell and inclusion: linear dimensions of the cell $L_{x}=L_{y}=$ $L_{z}\equiv L$, linear dimensions of the inclusion $l_{a}$ and $l_{b}$, their shapes, Young moduli of the polymer matrix ($E_{m}$) and the inclusion ($E_{i}$), and their Poisson ratios ($\nu_{m}$ and $\nu_{i}$, respectively). These dependences are omitted in the expression for $W_{el}$, but are taken into account implicitly.

As was stated above, in this work we consider three main “modes” of the cell response: inclusion rotation, inclusion translation, and cell deformation. Each of these modes has significantly different stress and strain distributions and different dependences of the cell response on the geometric parameters of the inclusion. Under the influence of magnetic field, a ferromagnetic inclusion becomes magnetized and rotates to align its magnetic moment with the direction of the field. The main motivation for studying this mode of inclusion displacement is the interaction between highly anisotropic magnetically hard particles and a uniform external magnetic field. Translational motion of inclusions occurs under the influence of non-uniform magnetic fields created both by external sources and other ferromagnetic inclusions. Magnetic interaction between filler particles in MAEs serves as the main motivation in this case. Finally, the deformation of the cell surrounding each inclusion can be a result of external mechanical load or movement of the surrounding inclusions. More detailed mathematical formulation of the mechanical problem is presented in Appendix A.

A quadratic approximation of the stored energy dependence on the displacement parameter can be used for small displacements. This corresponds to the case of linear elasticity and leads to simple and easy to analyze expressions for the cell response.

For the case of inclusion rotation, elastic energy of the cell can be expressed in the following way using linear approximation:

(4) $$W_{el,lin}\left(\theta ,\varphi ,\vec{\delta }\right)=\frac{1}{2}E_{r}{\left(\theta_{0},\varphi_{0}\right)\cdot \left(\theta -\theta_{0}\right)}^{2},$$

For the case of translational motion of the inclusion, elastic energy of the cell can be expressed in the following way using linear approximation:

(5) $$W_{el,lin}\left(\theta ,\varphi ,\vec{\delta }\right)=\frac{1}{2}\left(E_{tx}\left(\theta_{0},\varphi_{0}\right)\delta_{x}^{2}+E_{ty}\left(\theta_{0},\varphi_{0}\right)\delta_{y}^{2}+E_{tz}\left(\theta_{0},\varphi_{0}\right)\delta_{z}^{2}\right),$$

The coefficients relating energy to inclusion displacement can be called effective elastic moduli. Here, it is assumed that the external field is directed along the Z axis, and the anisotropic shape of the inclusion leads to translation elastic modulus having three components: $E_{tx}$, $E_{ty}$, and $E_{tz}$. These effective moduli and the effective rotation modulus $E_{r}$ depend on the initial position of the inclusion inside the cell. Deformation of the cell caused by external forces can also be expressed in terms of the linear approximation. In the case of uniaxial deformation, the energy dependence on the cell elongation/contraction has the following form:

(6) $$W_{el,lin}\left(\varepsilon_{cell}\right)=\frac{1}{2}E_{d}\left(\theta_{0},\varphi_{0}\right)\varepsilon_{cell}^{2},$$

Here, $E_{d}$ is the effective elastic modulus and it can characterize stiffening of the cell caused by the presence of hard filler in the composite, and $\varepsilon_{cell}$ is the relative elongation/contraction of the cell. Strictly speaking, each effective modulus depends on $\varepsilon_{cell}$ due to cell deformation creating different geometric configurations. We refer to $E_{r}$, $E_{tx}$, $E_{ty}$, $E_{tz}$, and $E_{d}$ as effective elastic moduli for the respective cell excitation mode, despite them not representing the real elastic modulus of the macroscopic sample, because they relate the stored elastic energy to the displacement parameter in each case.

Additionally, nonlinearity can be introduced in a simple way via the dependence of the effective moduli on the displacement parameters θ, δ and $\varepsilon_{cell}$: $E=E\left(\theta_{0},\varphi_{0},\theta ,\delta ,\varepsilon_{cell}\right)$. The effective moduli increase with increasing displacement in nonlinear fashion, and while this increase is insignificant for small displacements, our calculations show that it can reach 20% for large displacements and r ~ 5.

This approach based on FEM modeling and energy minimization allows us to calculate both local characteristics of the composite (stress and strain) and the characteristics of the cell as a whole (elastic modulus) while reducing calculation times compared to a full-field approach. This is important for studies of MAEs with anisometric filler on mesoscopic scale due to the need to consider a lot more filler configurations than in the case of spherical filler particles. Additional parameters include particle shapes, sizes, and their spatial orientation.

## 3. Model Verification

In order to verify the model, we compared the results obtained using our FEM calculations with analytical predictions of two separate theoretical approaches to modeling the mechanical properties of composite materials. These models describe specific cases of cell deformation and inclusion rotation.

### 3.1. Elastic Modulus of Polymer Composites with a Small Fraction of Spherical Particulate

One of the most widely known models of composite elastic properties is the model proposed by Guth and Gold [85] that was based on Einstein’s approach to describing the viscosity of a spherical colloidal particle suspension [86]. This approach can be applied to spherical particles dispersed in a solid polymer matrix and results in a general dependence of a physical quantity q that characterizes the composite with a filler particle volume concentration c:

(7) $$q\left(c\right)=q_{0}\left(1+\alpha_{1}c+\alpha_{2}c^{2}+\alpha_{3}c^{3}+\ldots \right),$$

Here, $q_{0}$ is the value of q in the absence of filler particles. For the case of uniaxial tension q=E, and Young’s modulus of the composite, represented by the power series, is most commonly reduced to a linear or quadratic polynomial or a Guth–Gold model [87]:

(8) $$E_{d}\approx E_{m}\left(1+2.5c+14.1c^{2}\right)=1+2.5c+14.1c^{2},$$

In our dimensionless finite-element model, the elastic modulus of the polymer matrix $E_{m}$ is taken as a unit of energy, so $E_{m}=1$. We can then compare FEM modeling with the following form of the Guth–Gold model:

(9) $$E_{d}/E_{m}=E_{d}=1+2.5c+14.1c^{2},$$

**Figure 2:**
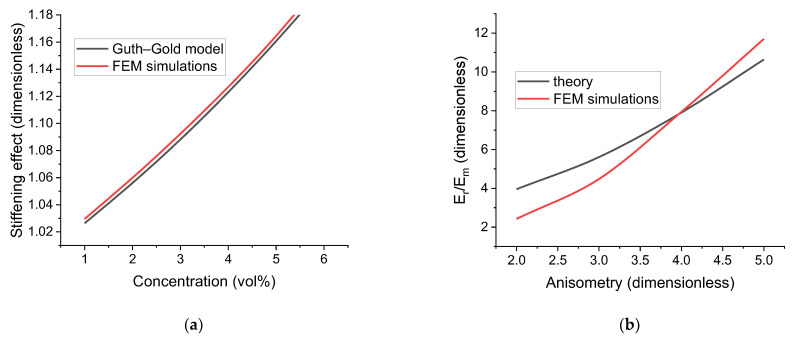
Comparison of FEM simulation results and analytical modeling: (**a**) uniaxial tension of a cell containing spherical inclusion: dependence of Young’s modulus of the composite on the spherical filler concentration; (**b**) rod-like inclusion rotation and comparison with the results of [84] for a filler concentration of 1% by volume: dependence of the relative effective rotation modulus on rod-like inclusion anisometry.

The comparison is shown in Figure 2a for 1%≤c≤6%. It can be seen that both the qualitative and quantitative agreement between the model and the results of FEM calculations are very good and demonstrate the consistency of our approach. The results of FEM modeling being higher than theoretical values can be attributed to both higher-order terms in the general model and the numerical errors related to the mesh density.

### 3.2. Displacements of a Rod in a Polymer Matrix

Explicit analytical models of the mechanical properties of polymer composites have been developed in a few notable works [84,88], and in most cases describe the influence of a single rigid inclusion and its movement on the material response. The inclusion can be said to be a probe that can help in studying the properties of the polymer matrix surrounding it. The main difference between these theoretical setups and our approach is the assumption of an infinite elastic medium, whereas the influence of the cell boundaries can be significant in our model. The existing theory describing displacements of a rod is based on solving the general field equation for displacement vector $\vec{u}$ in a linear, homogeneous, and isotropic elastic medium characterized by the Poisson ratio ν:

(10) $$\nabla \left(\nabla \cdot \vec{u}\right)+\left(1-2\nu \right)\nabla^{2}\vec{u}=0,$$

The fundamental solution of this equation can be used to obtain the disturbance created by the inclusion movement induced by an external force. The solution naturally depends on the type of the inclusion movement and on the inclusion shape. The work [84] considers an elongated single-domain ferromagnetic cylindrical inclusion rotating under the influence of a uniform external magnetic field. The mechanical energy stored in the medium is proportional to $\theta^{2}$, and the effective modulus can then be expressed as follows:

(11) $$E_{theory}=\frac{8r^{2}\left(1-\nu \right)G_{m}V_{i}}{3\left(3-4\nu \right)\ln r}=\frac{\pi r^{3}\left(1-\nu \right)E_{m}D^{3}}{3\left(3-4\nu \right)\left(1+\nu \right)\ln r}\approx \frac{\pi D^{3}r^{3}}{9\ln r}=\frac{4V_{i}r^{2}}{9\ln r},$$

Here, D is the diameter of the base of the cylinder and $G_{m}$ is the shear modulus of the polymer matrix. The corresponding results obtained using FEM modeling show that for lower values of r the theory presented above provides a higher effective modulus value than our calculations while for higher values of r the reverse is true (see Figure 2b). Two important factors to consider in this case are the influence of cell boundaries and the presence of a logarithmic function in the denominator. The logarithmic function can lead to overestimation of the modulus value for r ~ 2 due to its slow growth rate and ln⁡r≤1 in that case. The polymer medium is considered to be infinite in [84] while in our case the boundaries affect the cell response. In our setup, the minimal distance between the inclusion and the cell boundaries decreases with an increasing value of r, and that, in turn, tends to increase the resulting effective modulus, which can explain the discrepancy for r ~ 5. The overall agreement between the theory and FEM modeling can be said to be satisfactory with the considerations above taken into account.

The comparative analysis shows that the proposed single-inclusion modeling approach for calculating elastic energy of the cell reproduces the analytical results obtained by other researchers with reasonable accuracy, and thus our model can be said to be consistent with prior theoretical studies.

## 4. Mechanical Modeling Results

FEM simulations of different deformation modes in a purely mechanical problem were carried out to obtain the dependences of the mechanical response of the cell on both the external forces and the geometric parameters of the inclusion: shape, dimensions, and initial position inside the cell. The dependences of the effective elastic moduli of the cell on the geometrical parameters of the inclusion and the filler concentration in the material were considered. The filler concentration c was varied from 1% to 5% by volume.

### 4.1. Inclusion Rotation

**Figure 3:**
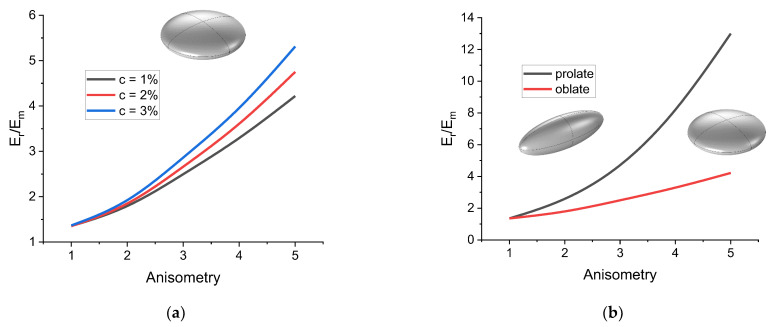
Dependences of the effective rotation modulus of a cell on the anisometry of the inclusion. (**a**) Comparison for different concentration values in the case of an oblate spheroid inclusion; (**b**) comparison for the cases of a prolate spheroid inclusion and an oblate spheroid inclusion (c=1%).

**Figure 4:**
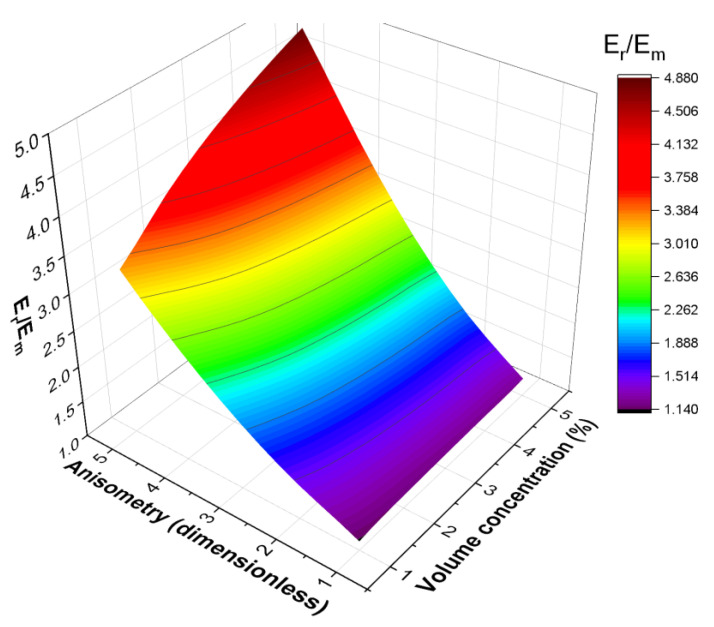
The dependence of the effective rotation modulus of a cell on the inclusion anisometry and filler volume concentration for a cell containing an oblate spheroid inclusion.

Figure 3 shows the dependences of the effective modulus of elasticity $E_{r}$ for the rotation of an oblate ellipsoidal inclusion on the parameter r for different values of the filler volume concentration. As the concentration increases, the dependence of the cell response on the geometric anisotropy of the inclusion becomes more pronounced. In this case, the dependence itself is non-linear, which can be explained by the dependence of the ellipsoid surface area on the length of its axes. Figure 4 compares the dependences of the effective modulus $E_{r}$ on the parameter r for the cases of prolate and oblate ellipsoidal inclusions. The presence of prolate filler particles leads to a more significant increase in the response of the cell than in the case of oblate filler. This means that for a given filler concentration, anisometry parameter, and filler magnetic properties, filler restructuring is more pronounced in composites containing oblate filler particles. This result is an agreement with experimental data [89].

A more general dependence $E_{r}/E_{m}=f\left(c,r\right)$ is shown in Figure 4. Figure 4 corresponds to the case of an oblate spheroid; however, the calculations show that the effective modulus increases more rapidly with the increase in r than with the increase in c for all considered inclusion shapes. A following function was considered for approximation of the $f\left(c,r\right)$ dependence in order to analyze the results from the point of view of scaling laws:

(12) $$E_{r}/E_{m}=1+E_{*}e^{sc}r^{\mu },$$

Here, $E_{*}$, s, and μ are the model parameters describing the growth of the effective modulus with c and r. Table 1 provides the values of these parameters for four different inclusion shapes: prolate and oblate spheroids and two types of cylinders (disk and rod). Cells containing elongated inclusions (prolate spheroids and rods) have the highest values of the effective modulus as well as the more pronounced dependences of the modulus on the anisotropy parameter r. The influence of the volume concentration is much weaker in comparison. Both ellipsoids and cylinders serve as rough approximations of real inclusion shapes, so neither can truly capture the behavior of particles and clusters with irregular shapes (which are frequently used in synthesis of MAEs), but it is clear that filler structures with elongated shapes can be used to create composites with high magnetically induced stiffness even at low filler concentrations. On the other hand, magnetically hard platelet filler structures can be used to create composites with a more pronounced response to uniform magnetic fields as the lower effective moduli allow for filler restructuring. The effective stiffness of the material in this case is still higher than the stiffness of a composite filled with spherical particles and aggregates.

All the given dependencies are calculated under the assumption of zero initial position parameters $\theta_{0}=0$, $\varphi_{0}=0$, ${\vec{\delta }}_{0}=0$. The calculations showed that small values of the initial position parameters do not lead to a noticeable change in the effective moduli. In particular, at low concentrations, the value of $\varphi_{0}$ practically does not affect the results, and varying $\theta_{0}$ between 0 and 90 degrees changes $E_{r}$ by less than 5%. However, the influence of the initial position parameters increases significantly for larger concentrations and larger values of r due to the decreasing distance between the inclusion and the cell boundaries. In a real material, this corresponds to two anisometric inclusions being close to each other, which leads to their relative orientations in space significantly affecting their movement. In this approach, the main parameters of the cell are filler volume concentration c and inclusion anisometry r.

### 4.2. Translational Motion of the Inclusion

**Figure 5:**
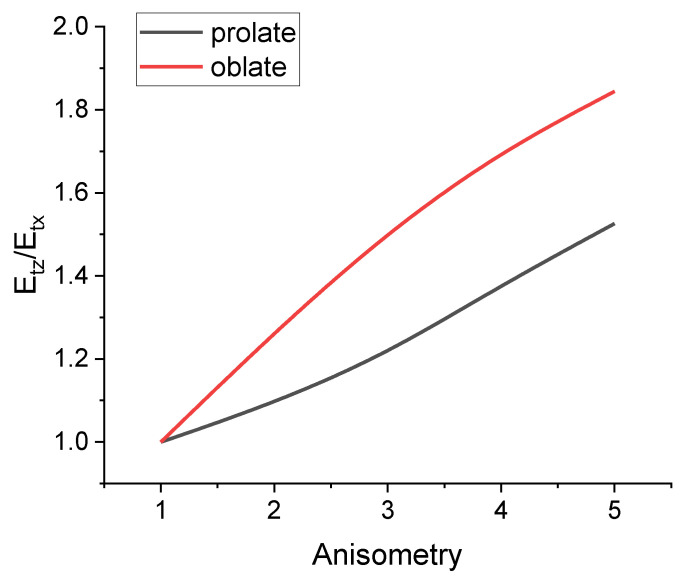
Dependence of the effective elastic modulus ratio (modulus for displacement along the minor axis $E_{tz}$ vs. modulus for displacement along the major axis $E_{tx}$) for the translational motion of a prolate spheroid and oblate spheroid inclusion on the anisotropy parameter. The filler concentration is 1%.

Figure 5 showcases the difference between the dependences of the effective elasticity moduli on the parameter r for the translational motion of a prolate and oblate spheroid inclusions along the major axis ($E_{tx}$) and along the minor axis ($E_{tz}$). The movement of a prolate inclusion along its minor axis obviously perturbs a larger area of the polymer matrix than movement along the major axis. The same holds true for an oblate inclusion. Following the same considerations, it is easy to see that the effective translational modulus is higher for the inclusion with an oblate spheroid shape.

**Figure 6:**
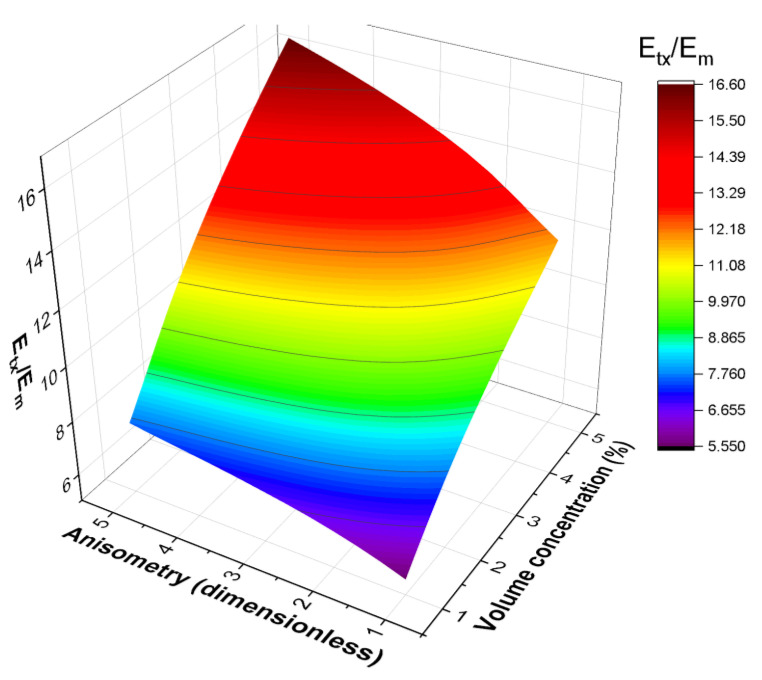
The dependence of the effective translation modulus of a cell on the inclusion anisometry and filler volume concentration for a cell containing an oblate spheroid inclusion and movement along the OX axis.

A more general dependence $E_{t}/E_{m}=f\left(c,r\right)$ is shown in Figure 6. Figure 6 demonstrates $f\left(c,r\right)$ for the case of an oblate spheroid inclusion and translation along the inclusion’s major axis. The dependence of $E_{tx}$ on the inclusion shape is similar to the case of $E_{r}$ discussed above with the exception of the growth with filler concentration being more pronounced for both elongated shapes than their platelet counterparts. For $E_{tz}$, the dependence on the inclusion shape is reversed: $E_{tz}$ is higher and increases more sharply for platelet inclusions. This is a natural result as the perturbations (and the resulting stress) in the polymer matrix are significantly higher for movement in the direction perpendicular to that of the major axis or the base of the inclusion.

### 4.3. Uniaxial Extension of the Cell

**Figure 7:**
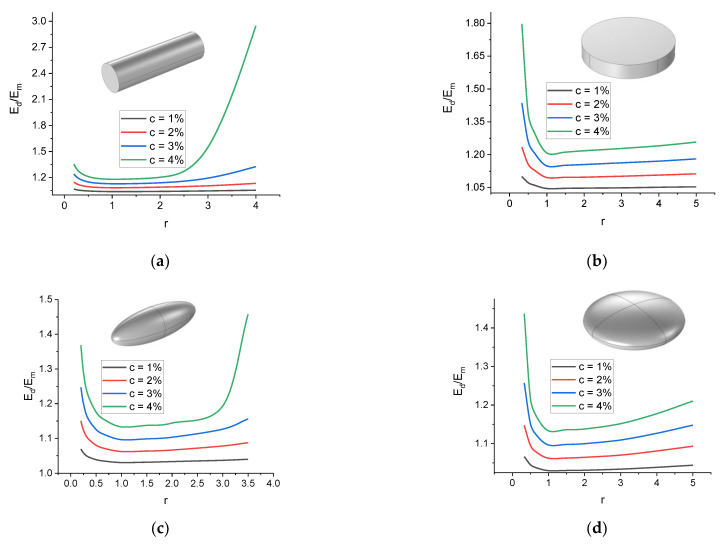
Examples of dependences of the effective tensile modulus of the cell on the inclusion anisotropy parameter at $\theta_{0}=0{}^{\circ}$ for inclusions of the following shapes: (**a**) rod-like cylinder; (**b**) disk-shaped cylinder; (**c**) prolate spheroid; (**d**) oblate spheroid.

Figure 7 shows an example of the dependence of the uniaxial tension elasticity modulus of a cell, $E_{d}$, on the parameter r for four different filler concentrations and different inclusions with tension applied along the Z axis. At higher concentrations, the nonlinearity of $E_{d}\left(r\right)$ dependence becomes more pronounced, which can be associated with an increase in the ratio of the cross-sectional area of the inclusion to the cross-sectional area of the cell for the direction perpendicular to the tensile load vector.

It should be noted that r<1 means that an elongated inclusion turns into a corresponding platelet inclusion with $\theta_{0}$ increasing by 90° and vice versa. Comparing two reference configurations for the cases of the inclusion’s largest section being perpendicular to the load force ($\theta_{0}=0{}^{\circ}$) and parallel to it ($\theta_{0}=90{}^{\circ}$) can provide us with information on how a composite would react to mechanical loads with different directions when anisotropic filler has a preferred alignment. In case of ferromagnetic inclusions, a magnetic field can be used to align them during the curing process. One can use the ratio of the effective moduli in the reference configurations mentioned above to describe the anisotropy of $E_{d}$:

(13) $$P=\frac{E_{d}\left(c,r,\theta_{0}=90{}^{\circ}\right)}{E_{d}\left(c,r,\theta_{0}=0{}^{\circ}\right)},$$

**Figure 8:**
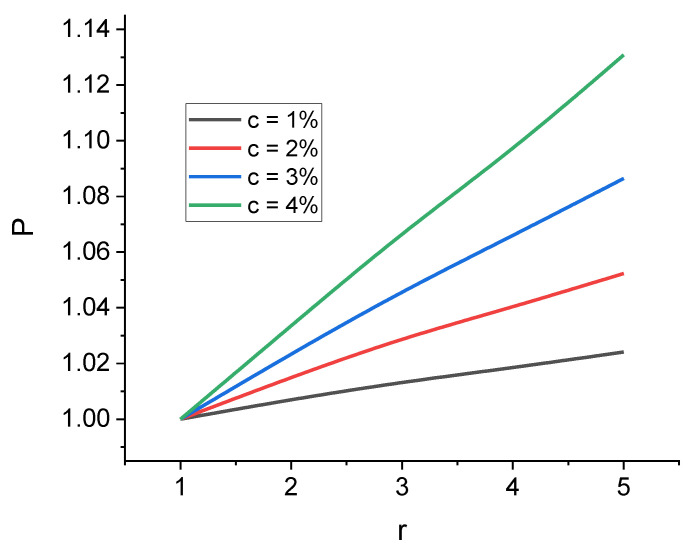
Dependences of the coefficient P on the inclusion anisotropy parameter r for oblate spheroid inclusions. Coefficient P is equal to the ratio of the tensile elastic modulus of the cell $E_{d}$ at $\theta_{0}=90{}^{\circ}$ to the modulus $E_{d}$ at $\theta_{0}=0{}^{\circ}$.

Figure 8 showcases the dependences of the ratio P on the inclusion anisotropy r and confirms the previously obtained results: $E_{d}$ is maximized when the largest section of the inclusion is parallel to the mechanical load vector. The difference between $E_{d}\left(c,r,\theta_{0}=90{}^{\circ}\right)$ and $E_{d}\left(c,r,\theta_{0}=0{}^{\circ}\right)$ is noticeable and can reach 13% for r ~ 5 in case of oblate particles and 46% for prolate ones.

**Figure 9:**
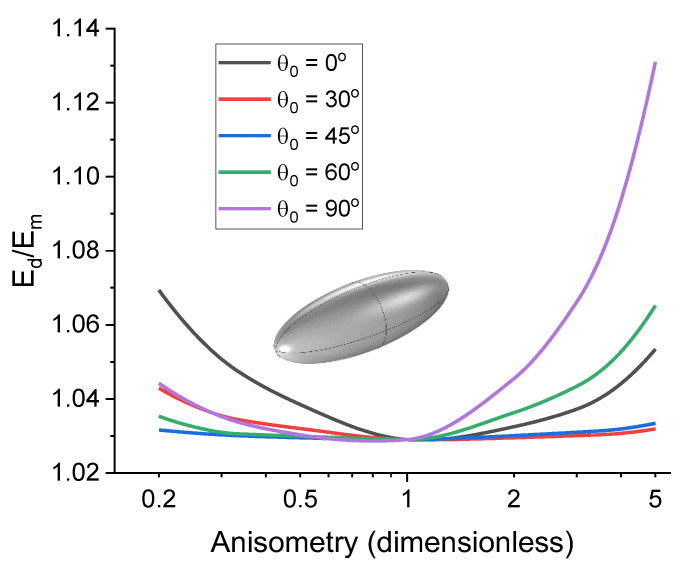
Demonstration of the influence of the initial inclusion rotation angle $\theta_{0}$ on the form of the effective tensile elastic modulus dependence on the inclusion anisometry parameter. A logarithmic scale has been introduced along the OX axis for clarity. Results are presented for a prolate spheroid inclusion and filler concentration of 1%.

Additionally, we evaluated the influence of the initial angle $\theta_{0}$ on the effective uniaxial tension modulus. The spatial orientation of inclusions in MAEs is random when no magnetic field is present, so it is important to consider how it affects the mechanical properties of the composite and what the average contribution of the initial spatial orientation would be. Figure 9 shows the dependences of the relative effective modulus $E_{d}/E_{m}$ on the inclusion anisometry for different values of the initial polar angle $\theta_{0}$. It is evident that the influence of the initial polar angle is the most prominent for the case of $\theta_{0}=90{}^{\circ}$, if the external load is aligned with OZ axis. A conclusion can be drawn that the effective elastic modulus of the cell has the highest value when the direction of the largest linear dimension of the inclusion is aligned with the direction of the external load. For inclusions with high anisometry, the effect of the initial polar angle on the response of the cell can reach 15%. On average, however, this value will be significantly lower.

The choice between using cylinders or ellipsoids for approximating the shape of the real inclusions in MAEs largely depends on both the filler particle material and the calculation algorithm. Ellipsoids have a smoother surface, but may also have singularities around their vertices. It can then be important to evaluate the differences in resulting effective moduli between the cells containing ellipsoidal and cylindrical inclusions with the same volume $V_{i}$. The ratio that describes this difference can be expressed as follows:

(14) $$D=\frac{E_{d,cyl}\left(c,r\right)}{E_{d,ell}\left(c,r\right)},$$

Here, $E_{d,cyl}$ and $E_{d,ell}$ are the effective moduli for uniaxial tension for cells containing a cylindrical and ellipsoidal inclusion, respectively. The dependences of ratio D on inclusion anisotropy r were calculated for different values of filler concentration. It is interesting to note that the behavior of D is different for cells with elongated and platelet inclusions, and the differences become more prominent with the increase in concentration. This is likely related to the effect the cell boundaries have on stress and strain in the cell, as the edges of elongated and platelet inclusions approach the boundaries in different ways with increasing r. The minimum distance between the inclusion and the boundaries is lower for elongated inclusions, so the boundary effects are more pronounced in that case. Our calculations show that D decreases monotonically and tends toward 1 with the increase in r for cells with platelet inclusions and increases with r for cells with elongated inclusions. For c≤5% and r≤5, the value of D does not exceed 1.12, so while it is important to take the shape of the inclusion into consideration, different idealized approximations of the real shape provide sufficiently similar results, so a geometrical configuration that is more convenient from the modeling point of view can be chosen.

**Figure 10:**
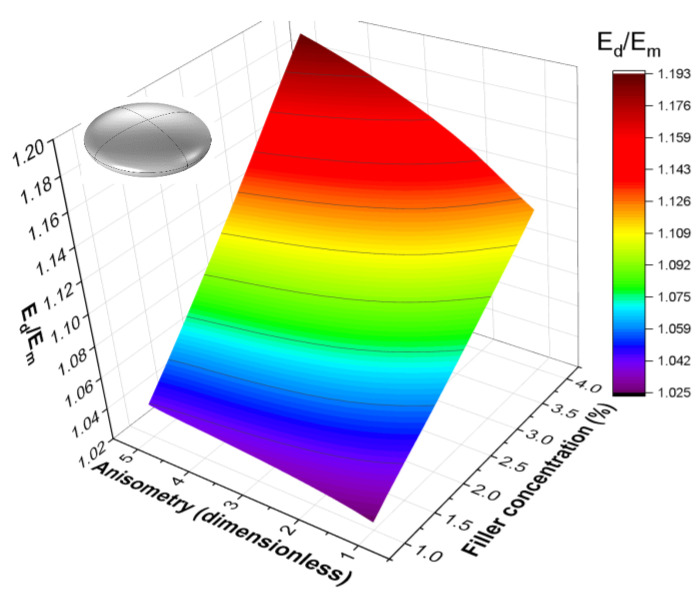
The dependence of the effective uniaxial tension modulus of a cell on the inclusion anisometry and filler volume concentration for a cell containing an oblate spheroid inclusion.

Figure 10 shows a more general form of the dependence $E_{d}=E_{d}\left(c,r\right)$ for an oblate spheroid inclusion. The approximation function used here to describe the dependence is the same as in the rotation and translation cases:

(15) $$E_{d}/E_{m}=1+E_{*}e^{sc}r^{\mu },$$

The values of the parameters $E_{*}$, s, and μ for $E_{d}$ are listed in the Table 2. The baseline parameter $E_{*}$ is higher for cells containing platelet inclusions; however, the growth parameters s and μ are higher for cells containing elongated inclusions. The highest relative effective modulus is achieved for cells with highly anisotropic cylindrical rods as inclusions.

Stress $\hat{\sigma }$ and strain $\hat{\varepsilon }$ tensor fields are the quantities that provide the most information about the mechanical state of the system. Detailed analysis of $\hat{\sigma }$ and $\hat{\varepsilon }$ is outside the scope of this paper due to a substantial amount of additional calculations required to perform it, but considering the distribution of stress and strain inside the cell can be useful for understanding the effect the cell boundaries have on the effective modulus. Stress and strain can be calculated using well known expressions of the finite strain theory:

(16) $$\begin{array}{c} \hat{\varepsilon }=\frac{1}{2}\left({\hat{F}}^{T}\hat{F}-\hat{I}\right)\\ \hat{\sigma }=\frac{1}{J}\hat{F}\frac{\partial \psi }{\partial \hat{F}}{\hat{F}}^{T} \end{array},$$

$\hat{I}$ denotes the identity tensor. We use this more general form of $\hat{\sigma }$ and $\hat{\varepsilon }$ due to the inherent nonlinearity of our model. Tensor quantities are difficult to represent visually (stress and strain fields have six independent components), so we make use of a specific scalar stress function in von Mises form:

(17) $$\sigma_{vM}=\sqrt{\frac{1}{2}\left({\left(\sigma_{11}-\sigma_{22}\right)}^{2}+{\left(\sigma_{22}-\sigma_{33}\right)}^{2}+{\left(\sigma_{33}-\sigma_{11}\right)}^{2}+6\left(\sigma_{12}^{2}+\sigma_{23}^{2}+\sigma_{13}^{2}\right)\right),}$$

**Figure 11:**
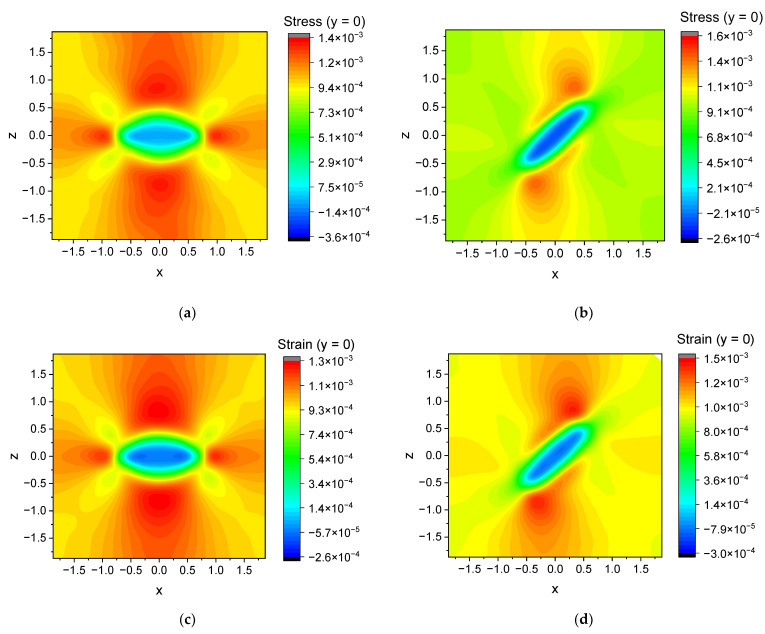
An example of a slice (y=0) of the von Mises mechanical stress (**a**,**b**) and the zz-component of the mechanical strain (**c**,**d**) distribution in a cell for the case of uniaxial tension. The distributions are shown for a cell with a filler concentration c=1%, an oblate spheroid inclusion, r=3, $\theta_{0}=0{}^{\circ}$, $\varphi_{0}=0{}^{\circ}$ (**a**,**c**) and r=3, $\theta_{0}=45{}^{\circ}$, $\varphi_{0}=0{}^{\circ}$ (**b**,**d**). The deformation is fixed at 0.1% of L.

Examples of the sections of stress distributions for y=0 are shown on Figure 11. The distributions are highly anisotropic. The maximum stress is concentrated near the particle surface. In accordance with previous conclusions, less characteristic stress is induced in cells with tilted particles.

## 5. Inclusion Rotation in External Magnetic Field

Using the results obtained in the previous sections it is possible to simulate the state of the unit cell under the influence of external magnetic field. This stationary state corresponds to the minimum of the total energy where the mechanical part of the energy is determined based on the purely mechanical FEM calculations. Let us consider the case of an anisotropic ferromagnetic inclusion that rotates in external uniform magnetic field $\vec{H}$. For the sake of simplicity, let us also assume that the inclusion is characterized by a single magnetic moment vector $\vec{m}$. The total energy of the cell in the presence of external magnetic field H can be expressed using the classic Stoner–Wohlfarth model and $W_{el}$ discussed in Section 2.2:

(18) $$W_{cell}=-mHcos\alpha -KV_{i}{sin}^{2}\left(\theta_{0}+\theta +\alpha \right)+W_{el}\left(\theta ,\varphi ,\vec{\delta }\right),$$

The first term here corresponds to Zeeman energy with α being the angle between $\vec{m}$ and $\vec{H}$ (or the Z axis). The second term represents the magnetic anisotropy energy with K being the magnetic anisotropy constant that is defined by the crystal structure of the inclusion material and $V_{i}$ being the inclusion volume. The magnetic anisotropy energy term in this model serves as a form of magneto-mechanical coupling as it determines the extent to which the rotation of the magnetic moment affects the geometric rotation of the inclusion. We assume that the easy magnetization axis is aligned with the long axis of the inclusion.

It is important to note that the external magnetic field H used here is the dimensionless magnetic field calculated relative to $\sqrt{E_{m}}$, where $E_{m}$ is the elastic modulus of a pure polymer matrix. One can show that units for H and $\sqrt{E_{m}}$ are the same when expressed in terms of basic CGS units. If the external magnetic field has a maximum magnetic flux value of 1 T (which corresponds to magnetic field strength of 10 kOe), then the maximum dimensionless field value for a material with $E_{m}=10$ kPa is around 31.62 and for the case of $E_{m}=100$ kPa the dimensionless field can only reach the strength of 10.

The magnetization M depends on the material used as ferromagnetic filler. There are two large groups of ferromagnetic materials: magnetically soft and magnetically hard materials [90]. Magnetically hard materials have high remanence, so if we consider filler with remanent flux $B_{r}$ higher than 1 T, then magnetization can be considered to be a constant: $M=M_{r}$. One of the most widely used fillers for MAEs is the NdFeB alloy [91] that can have remanence of around 1.3 T. For the case of $E_{m}=10$ kPa, this corresponds to dimensionless M≈41.11 and for the case of $E_{m}=100$ kPa, to M=13. Magnetically soft materials, on the other hand, have low remanence and thus cannot be described using constant magnetization for any external field. To express the dependence of magnetization on external magnetic field for fillers of this group we use the Fröhlich–Kennelly model [92]:

(19) $$M\left(H\right)=\frac{M_{s}H}{H+M_{s}/\chi_{0}},$$

Here, $M_{s}$ is the saturation magnetization and $\chi_{0}$ is the linear magnetic susceptibility of the material. For another commonly used ferromagnetic filler, pure iron, these quantities are as follows: $M_{s}\approx 1.7\times 10^{6}$ A/m and $\chi_{0}\approx 1100$.

Finally, the anisotropy constant K is a magnetocrystalline constant that describes the degree of magnetic anisotropy: the higher the anisotropy constant is, the more energetically unfavorable the state with magnetization not corresponding to the preferred direction is. For NdFeB, the anisotropy constant is $K=4.9\times 10^{6}$ J/m^3^, which corresponds to the dimensionless values of 490 and 49 for $E_{m}=10$ kPa and $E_{m}=100$ kPa, respectively. For pure iron, $K=4.8\times 10^{4}$ J/m^3^, so the dimensionless magnetic anisotropy constant is 4.8 and 0.48 for the cases of $E_{m}=10$ kPa and $E_{m}=100$ kPa, respectively.

Let us then consider the limiting cases of materials with K=0 (infinitely soft) and K=∞ (infinitely hard). The former case corresponds to the free rotation of the magnetic moment in an external magnetic field. One can expect that the magnetic field in this case will not affect the particle position and the resulting modulus of the composite assuming interactions between the particles at low filling concentrations are negligible. The latter case corresponds to the inclusion rotation strictly following its magnetic moment, and the resulting equilibrium angle will be defined by an interplay between elastic energy and Zeeman energy. For the realistic cases of 0<K<∞, the inclusion behavior is defined by the ratio between the anisotropy constant and the effective elastic modulus $A=K/E_{r}$, as will be shown further.

**Figure 12:**
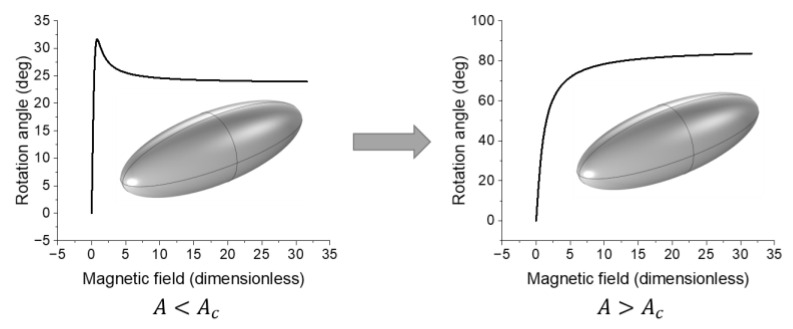
Different types of inclusion rotations in the presence of external magnetic field that can be determined by the ratio of the magnetic anisotropy energy to stored mechanical energy $A=K/E_{r}$.

As it was noted previously, if the energy of magnetic anisotropy is not significant enough, the restructuring due to inclusion rotation is going to be less prominent. This is especially relevant for magnetically soft filler. Analysis shows that within the framework of the model used in this study, the ratio of magnetic anisotropy constant A to effective elastic modulus $E_{r}$ can represent the ratio of magnetic anisotropy energy to elastic energy and serve as a characteristic of the $\theta \left(H\right)$ dependence. The value of $A=K/E_{r}$ that corresponds to the transition from monotonically increasing $\theta \left(H\right)$ to a function with an extremum (or the other way around) can be denoted as $A_{c}$. Thus, if $A<A_{c}$, then $\theta \left(H\right)$ has a maximum and increasing the strength of the external magnetic field past the value corresponding to that maximum does not lead to further filler restructuring. On the other hand, if $A>A_{c}$, then increasing the magnetic field always creates stronger material response. Figure 12 demonstrates this result by showing the difference in inclusion movement between the two cases discussed above. The maximum in the $\theta \left(H\right)$ dependence arises when absolute values of all three terms of $W_{cell}$ are comparable to each other. This is realized if $A<A_{c}$. In that case, the anisotropy energy is significant enough to force the inclusion to follow its magnetic moment. As Zeeman energy increases with the external magnetic field, the coupling between the magnetic moment and the geometric axis of the inclusion becomes weaker, resulting in partial relaxation of the inclusion (the value of θ decreases). If $A>A_{c}$, magnetic anisotropy can always keep up with Zeeman energy leading to monotonically non-decreasing $\theta \left(H\right)$.

In order to calculate the value of $A_{c}$, let us consider the conditions that lead to $\theta \left(H\right)$ having an extremum. The obtained function $\theta \left(H\right)$ minimizes the energy functional $W_{cell}$, thus, the following holds true:

(20) $$\frac{\partial W}{\partial \alpha }=0,\;\frac{\partial W}{\partial \theta }=0\overset{\alpha =\alpha \left(\theta \right)}{\Rightarrow }f\left(H,\theta \left(H\right)\right)=0,$$

This means that $\theta \left(H\right)$ can be analytically expressed as an implicit function. The potential extremum point can then be obtained via the following system of nonlinear equations:

(21) $$\left\{\begin{array}{l} f\left(H,\theta \left(H\right)\right)=0\\ \theta '\left(H\right)=-f_{H}/f_{\theta }=0 \end{array},\right.$$

This system was solved numerically for different sets of physical parameters $\left(M,K,E_{r}\right)$ and different initial values of H and θ. Our calculations show that for 0<M<100, 0<K<500, and $1<E_{r}<10$, the function $\theta \left(H\right)$ can only have a maximum when A<1.5. Thus, $A_{c}\approx 1.5$ for both cells containing magnetically soft and magnetically hard fillers. This value is slightly reduced for materials with low saturation magnetization. Let us compare two magnetically soft materials: for one, the saturation magnetization is equal to that of pure iron, and for the other, it is 10 times less than that of pure iron. The value of $A_{c}$ of the second material is only ~2.1% less than that of the first material. The same comparison for magnetically hard materials pre-magnetized to saturation with a baseline corresponding to NdFeB leads to ~5.6% difference in $A_{c}$ values. The expected deviation of $A_{c}$ from 1.5 is less than 5% for realistic cases of ferromagnetic fillers used in MAE synthesis. This means that in order to synthesize a composite with the highest response to magnetic field for low filler concentration values, the ferromagnetic filler material should be characterized by a magnetic anisotropy constant $K>1.5E_{r}$ (in CGS units). For example, oblate filler particles with r=5 at concentration c=5% by volume should have magnetic anisotropy constant $K>7.235\times 10^{4}$ J/m^3^, if they are dispersed in a polymer matrix with a Young’s modulus of 10 kPa. This value of magnetic anisotropy constant is 72% higher than that of pure iron. The corresponding value for the case of prolate filler particles is $3.445\times 10^{5}$ J/m^3^. In order for A to be equal to $A_{c}$ for filler particles with magnetic properties similar to pure iron in this case, the Young’s modulus of the matrix has to be equal to 5.8 kPa and 1.2 kPa for the oblate and prolate spheroid particles, respectively. It then follows that magnetically hard fillers with oblate shapes are more suitable for the purpose of creating MAEs with the highest degree of filler restructuring at low filler concentrations.

**Figure 13:**
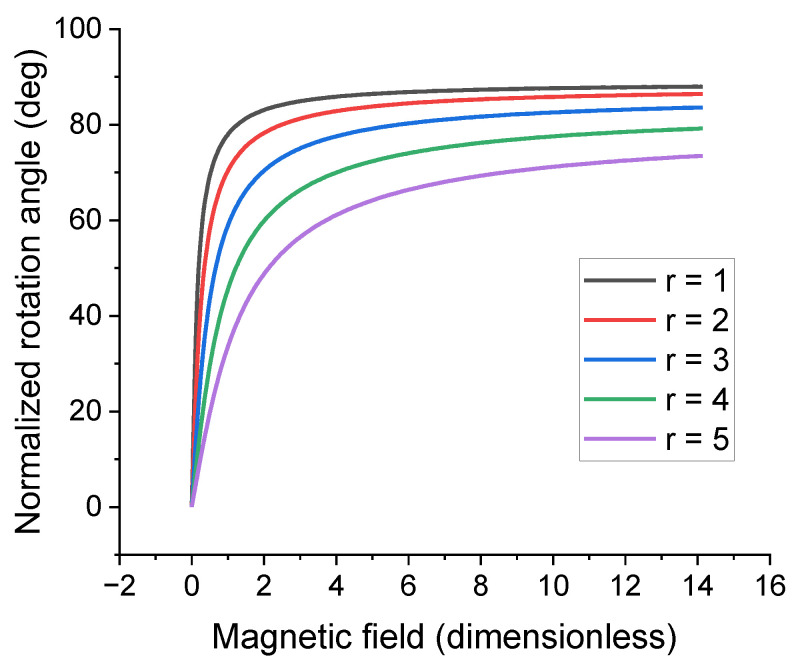
Dependence of the rotation angle of a prolate spheroid inclusion on the magnitude of the external magnetic field for various values of the anisotropy parameter. The filler concentration is 1% by volume. The magnetic anisotropy constant K is set to $4.9\times 10^{6}$ J/m^3^ corresponding to NdFeB material properties.

Figure 13 shows an example of the dependence of the resulting polar rotation angle of a disk-like inclusion on the magnitude of the dimensionless magnetic field assuming filler volume concentration to be 1% by volume. The general form of the dependence depicted in Figure 13 is determined by the anisotropy constant value and the magnetization of the inclusion. The magnetic moment of a particle in this model is considered to be independent of its shape. The rotation angle reaches saturation when the angle between the magnetic moment of the particle and the external field is minimized, and the magnetic anisotropy energy and the elastic energy of the cell cancel each other out.

The higher the anisotropy parameter r is, the less rotational freedom the inclusion has due to the effective elastic modulus of the cell increasing with increasing r. Additionally, the inclusion volume is kept constant throughout the parametric sweep. The modeling data suggests that the $\theta \left(H\right)$ dependence can be described using a sigmoid function for any given r while the $\theta \left(r\right)$ dependence can be described with a power function for any given H. Therefore, it was decided to use the following scaling function to describe the $\theta =\theta \left(H,r\right)$ dependence:

(22) $$\theta \left(H,r\right)=\theta_{*}{\left(r_{0}-r\right)}^{\nu }\frac{H/H_{*}}{\sqrt{1+{\left(H/H_{*}\right)}^{2}}},$$

**Figure 14:**
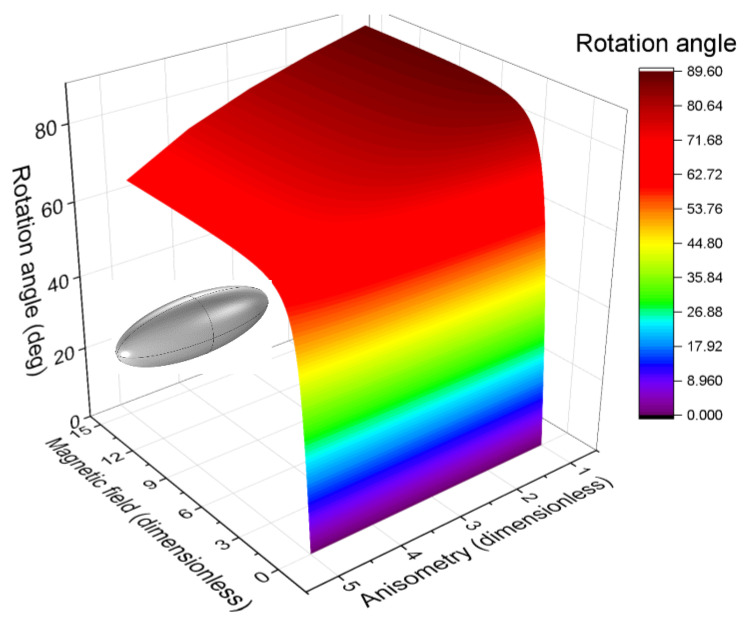
The dependence of the inclusion rotation angle on the inclusion anisometry and external magnetic field for a cell containing a magnetically hard prolate spheroid inclusion with filler concentration of 1% by volume.

An example of a $\theta =\theta \left(H,r\right)$ surface is shown in Figure 14. It corresponds to a NdFeB inclusion with a prolate spheroid shape and filler volume concentration c=1%.

The $\theta \left(H\right)$ dependence determines the degree of filler restructuring and, through it, the stiffening effect. As such, it is important to know the characteristics of the filler one needs to disperse in a given polymer matrix to achieve the highest response of the resulting composite to external magnetic field. In particular, the shape of the $\theta \left(H\right)$ curve indicates whether or not strong magnetic fields cause the strongest composite response.

## 6. Conclusions

In this study, we have formulated, tested, and analyzed a numerical dimensionless model of a magnetoactive elastomer volume element containing ferromagnetic inclusions with geometric and magnetic anisotropy. The model employs a single-inclusion cell, describing the cell’s reaction to an external magnetic field within a simple approximation framework, disregarding magnetic interactions between the different inclusions. Those inclusions can represent micro-scale ferromagnetic filler or pre-magnetized particle aggregates that formed under the influence of external magnetic field before the start of the simulation. The main purpose of this work was to study the effects of the inclusion’s shape and magnetic characteristics on the cell response to external forces by dividing the problem into two parts: purely mechanical and magneto-mechanical with pre-determined mechanical properties. Mechanical aspects of the modeling were tackled through finite-element method simulations, while the magnetic response of an inclusion with anisotropic ferromagnetic properties was addressed using the Stoner–Wohlfarth model.

First, we examined the mechanical response of cells to external stimuli, considering three deformation modes: particle rotation, particle translation, and cell elongation. The mechanical aspect of the problem has significance on its own, as it can be applied to any composite material that contains filler inclusions. Our analysis of rotational and translational motion demonstrated how particle shape influences restructuring within the polymer matrix, enabling us to quantify the effects of particle shape and anisotropy. A general scaling expression for the anisometry and concentration dependence of the effective rotational modulus was derived, and the scaling exponents for different inclusion shapes were calculated by fitting FEM results. The results indicated that for the same level of anisometry, oblate filler particles have a lower effective rotational modulus compared to prolate particles. This means that the oblate particles can rotate under the influence of smaller forces than the prolate particles, resulting in more effective restructuring in the polymer matrix for the same applied force. It was shown that translational motion in the direction perpendicular to the major axis of the inclusion is more difficult than along it due to greater perturbations of the matrix. Calculating the effective rotational and translational moduli, and their relative values for different shapes and anisometry of inclusions, is crucial for the further development of coarse-grained models in molecular dynamics simulations of MAEs containing anisometric particles.

The cell uniaxial tension analysis enabled us to estimate the elastic modulus of the composite material for different inclusion shapes and anisometries at low filling degrees. The study demonstrated that anisometric inclusion orientation relative to the external force direction significantly affects the cell elastic modulus. It is maximized when the inclusion’s largest linear dimension aligns with the direction of the external load. For high anisotropy, the modulus value drops by 15% if the particle major axis is oriented perpendicular to the external force. These results are in agreement with recent experimental data obtained for anisotropic MAEs that were synthesized in magnetic fields and thus acquired an oriented structure of anisometric magnetic particles [83]. The impact of the inclusion shape was analyzed within the developed approach and the scaling laws for the cell elastic modulus were found depending on the particle anisometry and concentration.

Subsequently, we obtained the dependences of the inclusion displacement on the external magnetic field and analyzed ferromagnetic properties of the inclusions required for achieving the highest degree of filler restructuring. Our findings indicate that even low concentrations of anisometric magnetic particles in a MAE can lead to considerable augmentation in elastic properties when a uniform magnetic field is applied.

The most intriguing discovery is the identification of two distinct particle behavior scenarios in a magnetic field, depending on the ratio of magnetic anisotropy energy to the elastic energy of the cell, characterized by the parameter $A=K/E_{r}$, where K and $E_{r}$ represent the anisotropy constant and effective rotational modulus, respectively. In the first scenario, which occurs when A is higher than a critical value $A_{c}$, the particle’s rotational angle increases steadily with the magnetic field, causing it to align with the field. In contrast, the second scenario occurs when $A<A_{c}$ and the magnetic anisotropy is not as strong. In this case, the particle initial rotation with the magnetic field is followed by the decoupling of the magnetic moment from its anisotropy axis and the decrease in the rotational angle.

The most important qualitative conclusions that can be drawn from the obtained results can be formulated as follows:

- For a given degree of filling, increasing the anisometry of the filler particles increases the modulus of the cell;
- At fixed anisometry, the effect of the particles on the elastic modulus of the cell is maximized when the orientation of the major axis of the inclusion is parallel to the applied mechanical force;
- The higher the anisometry of the inclusion, the greater the force required to rotate it; however, for the same anisometry, oblate particles rotate more easily than prolate particles;
- The effect of the external magnetic field on the rotation of the inclusion increases with the magnetic anisotropy of the inclusion.

Additionally, we substantiated the applicability of this approach for MAEs with low (around 5% by volume) filler concentrations by comparing the simulation results with two previously published analytical models. It should be noted that even for such low concentrations, the influence of the shape and dimensions of an anisotropic inclusion on the cell response is significant and allows one to increase the effective elastic moduli of the cell by up to two orders of magnitude compared to the spherical inclusions case. The obtained results underscore the efficacy of incorporating anisometric magnetic particles in MAE composites to fabricate devices with highly tunable mechanical properties. The modeling of interactions between anisometric inclusions and the averaging of results to describe processes on larger scales are the focus of our current research. As most limitations of the presented model are rectifiable without altering the core components, we deduced that this model represents a step towards creating a robust and universally applicable numerical tool for scrutinizing the local restructuring in MAEs.

## Figures and Tables

**Table 1:** Values of the modeling parameters $E_{*}$, s, and μ for the $E_{r}/E_{m}=f\left(c,r\right)$ dependence calculated for different inclusion shapes.

Parameter	Prolate Spheroid	Oblate Spheroid	Rod-like Cylinder	Disk-like Cylinder
$E_{*}$	$1.7092\times 10^{-1}$	$1.4455\times 10^{-1}$	$1.1252\times 10^{0}$	$1.2013\times 10^{0}$
*s*	$1.2892\times 10^{-1}$	$1.2968\times 10^{-1}$	$1.0917\times 10^{-1}$	$3.7523\times 10^{-2}$
*μ*	2.2644	1.6628	1.2759	0.631

**Table 2:** Values of the modeling parameters $E_{*}$, s, and μ for the $E_{d}/E_{m}=f\left(c,r\right)$ dependence calculated for different inclusion shapes.

Parameter	Prolate Spheroid	Oblate Spheroid	Rod-like Cylinder	Disk-like Cylinder
$E_{*}$	$1.7092\times 10^{-1}$	$1.4455\times 10^{-1}$	$1.1252\times 10^{0}$	$1.2013\times 10^{0}$
*s*	$1.2892\times 10^{-1}$	$1.2968\times 10^{-1}$	$1.0917\times 10^{-1}$	$3.7523\times 10^{-2}$
*μ*	2.2644	1.6628	1.2759	0.631

## Data Availability

Data are contained within the article.

## Appendix A

In the case of hyperelastic media, the boundary value problem of elasticity is equivalent to a minimization problem for the total energy of the system W:

(A1) $$W=\int_{V}\psi \left(\vec{u}\right)dV-\int_{V}\left(\vec{f},\vec{u}\right)dV-\int_{\Gamma }\left(\vec{b},\vec{u}\right)dS,$$

Here, ψ is the stored strain energy density function, $\vec{u}$ is the displacement field inside the cell, $\vec{f}$ represents the conservative body forces per unit of volume, $\vec{b}$ represents the conservative boundary forces per unit of area, V is the domain in which the problem is being solved, and Γ is the external boundary of V. The variational formulation of the minimization problem can be written in the following way:

$L\left(u,v\right)={\left.\frac{dW\left(u+\epsilon v\right)}{d\epsilon }\right|}_{\epsilon =0}=0$ for all test functions v that satisfy the boundary conditions of the boundary value problem and belong to the same differentiability class as u.

The model presented in this work allows for simulation of different mechanical problems by simply adding or removing load terms to the energy functional W or by changing the boundary conditions. The three main cases considered here inclusion rotation, translational motion of the inclusion, and uniaxial cell tension. These are the three elementary excitations of the cell that characterize its mechanical response to external influence. The mathematical expressions governing the FEM problems in each case are presented below.

1. Pure rotation

Boundary conditions:

(A2) $${\left.u\right|}_{\Gamma }=0,$$

Here, u denotes all components of the displacement vector.

Energy functional of the cell:

(A3) $$W_{mech,rot}=\int_{V\backslash V_{i}}\psi_{m}dV+\int_{V_{i}}\psi_{i}dV-\int_{V_{i}}\left(\vec{T},\vec{u}\right)dV\to min,$$

Here, $V_{i}$ is the inclusion volume, $\psi_{m}$ and $\psi_{i}$ are strain energy densities for the polymer matrix and the inclusion, respectively, and $\vec{T}=\vec{T}\left(x,y,z\right)$ is the torque field acting on the inclusion. Its components are defined as follows:

(A4) $$\begin{array}{c} T_{x}=-sgn\left(x\cos\varphi \cos\theta +y\sin\varphi +z\cos\varphi \sin\theta \right)T\cos\varphi \sin\theta \\ T_{y}=-sgn\left(x\cos\varphi \cos\theta +y\sin\varphi +z\cos\varphi \sin\theta \right)T\sin\varphi \sin\theta \\ T_{z}=sgn\left(x\cos\varphi \cos\theta +y\sin\varphi +z\cos\varphi \sin\theta \right)T\cos\theta \end{array},$$

This form of $\vec{T}$ is a vector field with a specified absolute value T that “follows” the inclusion’s rotation and is always perpendicular to the plane Π containing the axis of rotation and the major axis of the inclusion. Additionally, it is antisymmetric with respect to a plane perpendicular to Π. The angles φ and θ are shown on Figure 1b.

(A5) $$\begin{array}{c} \psi_{m}=\frac{G_{m}}{2}\left(I_{1}-3\right)-G_{m}ln\left(J\right)+\frac{\lambda_{m}}{2}{ln}^{2}\left(J\right)\\ \psi_{i}=\frac{G_{i}}{2}\left(I_{1}-3\right)-G_{i}ln\left(J\right)+\frac{\lambda_{i}}{2}{ln}^{2}\left(J\right) \end{array},$$

Here, $G_{m}$ and $G_{i}$ are shear moduli of the polymer matrix and the inclusion, $\lambda_{m}$ and $\lambda_{i}$ are the Lame parameters of the polymer matrix and the inclusion. In the case of linear elasticity approximation, the energy of the cell can be expressed as follows:

(A6) $$\begin{array}{c} W_{mr}={k_{r}\cdot \left(\theta -\theta_{0}\right)}^{2}\\ \frac{1}{V}W_{mr}={\frac{1}{2}E_{r}\cdot \left(\theta -\theta_{0}\right)}^{2} \end{array},$$

θ is the angle of the inclusion rotation, $\theta_{0}$ is the angle of the initial spatial orientation of the inclusion in the cell, and $k_{r}$ is the effective stiffness parameter.

2. Pure translation

Boundary conditions:

(A7) $${\left.u\right|}_{\Gamma }=0,$$

Energy functional of the cell:

(A8) $$W_{mech,tr}=\int_{V\backslash V_{i}}\psi_{m}dV+\int_{V_{i}}\psi_{i}dV-\int_{V_{i}}\left(\vec{F},\vec{u}\right)dV\to min,$$

Here, $\vec{F}$ is the force acting on the inclusion. In the case of linear elasticity approximation, the energy of the cell can be expressed as follows:

(A9) $$\begin{array}{c} W_{mt}={k_{tx}\cdot ∆x}^{2}+{k_{ty}\cdot ∆y}^{2}+{k_{tz}\cdot ∆z}^{2}\\ \frac{1}{V}W_{mt}=\frac{1}{2}{E_{tx}\cdot ∆x}^{2}+\frac{1}{2}{E_{ty}\cdot ∆y}^{2}+\frac{1}{2}{E_{tz}\cdot ∆z}^{2} \end{array},$$

∆x, ∆y, and ∆z are inclusion center of mass displacements along OX, OY, and OZ axes, respectively.

3. Pure deformation

Boundary conditions:

(A10) $$\begin{array}{cc} {\left.u\right|}_{\Gamma_{z+}}=∆L/2 & {\left.u\right|}_{\Gamma_{z-}}=-∆L/2\\ {\left.u\right|}_{\Gamma_{x+}}={\left.u\right|}_{\Gamma_{y+}}=-\frac{1}{2}\left(\frac{L^{1+\nu }}{{\left(L+∆L\right)}^{\nu }}-L\right) & {\left.u\right|}_{\Gamma_{x-}}={\left.u\right|}_{\Gamma_{y-}}=\frac{1}{2}\left(\frac{L^{1+\nu }}{{\left(L+∆L\right)}^{\nu }}-L\right) \end{array},$$

$\Gamma_{z+}$, $\Gamma_{z-}$, $\Gamma_{x+}$, $\Gamma_{x-}$, $\Gamma_{y+}$, $\Gamma_{y-}$ are the top, bottom, right, left, front, and back faces of the cell, respectively, ∆L is the prescribed displacement on the boundary, L is the linear dimension of the cell, and ν is the Poisson’s ratio of the polymer matrix.

Energy functional of the cell:

(A11) $$W_{mech,def}=\int_{V\backslash V_{i}}\psi_{m}dV+\int_{V_{i}}\psi_{i}dV\to min$$

In the case of linear elasticity approximation, the energy of the cell can be expressed as follows:

(A12) $$\begin{array}{c} W_{md}={k_{d}\cdot \left(∆L\right)}^{2}\\ \frac{1}{V}W_{md}={\frac{1}{2}E_{d}\cdot \left(\frac{∆L}{L}\right)}^{2} \end{array}$$
